# Reserves of Calcium, Copper, Iron, Potassium, Magnesium, Manganese, Sodium, Phosphorus, Strontium and Zinc in Goose Egg Yolk during Embryo Development

**DOI:** 10.3390/ani13121925

**Published:** 2023-06-08

**Authors:** Maghsoud Besharati, Leila Fathi, Saeid Amirdahri, Zabihollah Nemati, Valiollah Palangi, Jose Manuel Lorenzo, Aristide Maggiolino, Gerardo Centoducati

**Affiliations:** 1 Department of Animal Science, Ahar Faculty of Agriculture and Natural Resources, University of Tabriz, Ahar 5451785354, Iran; leilafathi9194@yahoo.com (L.F.) s.amirdahri60@gmail.com (S.A.) znnemati@yahoo.com (Z.N.); 2 Department of Animal Science, Faculty of Agriculture, Ege University, Izmir 35100, Türkiye; valiollah.palangi@ege.edu.tr; 3 Centro Tecnológico de la Carne de Galicia, Avd. Galicia 4, Parque Tecnológico de Galicia, 32900 Ourense, Spain; jmlorenzo@ceteca.net; 4 Facultad de Ciencias de Ourense, Área de Tecnología de los Alimentos, Universidade de Vigo, 32004 Ourense, Spain; 5 Department of Veterinary Medicine, University of Bari, 70010 Bari, Italy; gerardo.centoducati@uniba.it

**Keywords:** incubation, yolk, minerals, goose egg

## Abstract

**Simple Summary:**

Several studies have reported the absolute weight and concentration of minerals in hen and turkey yolks during incubation. However, there is limited information about some minerals in goose egg yolks during the incubation period. The incubation period of goose chicks is 30 days, and the chicks emerge after this period. As a result of mineral transfer between the ovary, yolk, albumin and shell, egg yolk mineral content increases during incubation. We studied whether the mineral reserves of goose egg yolk changed, and whether mineral deficiency in goose embryos resulted in skeletal, cardiovascular and immune weakness. According to the present study, except for calcium, the content of all the examined minerals decreases during incubation.

**Abstract:**

This study evaluated the changes in calcium (Ca), copper (Cu), iron (Fe), potassium (K), magnesium (Mg), manganese (Mn), sodium (Na), phosphorus (P), strontium (Sr) and zinc (Zn) values in goose egg yolk during the incubation period. This study was conducted on a completely random selection using 200 fertile eggs from a local flock of geese. A selection of 30 fertile eggs were randomly sampled on days 0, 9.5, 19.5, 25 and 30 of incubation (total: 150 eggs), and the yolks of 5 eggs in each replicate were mixed together and considered as one replicate (six replicates in each incubation period). The mineral content of the yolks was measured using the inductively coupled plasma (ICP) spectroscopy method. The results of this study show that, during the incubation of goose eggs from 0 to 30 days of embryo growth, except for Ca, the yolk content (absolute weight) of all the measured minerals, including Cu, Fe, K, Mg, Mn, Na, P, Sr and Zn, on day 30 of incubation were statistically significantly lower than on day 0 of incubation. Additionally, the yolk concentrations of Fe, K, Mg, Na, P and Zn declined, the yolk concentrations of Ca and Sr increased, and the yolk concentrations of Cu and Mn were unchanged on day 30 compared to the first day of incubation. The results of the current study demonstrate that goose egg yolks’ mineral concentrations change in absolute value during the incubation period, which could be the basis for further studies on chick feeding during the embryonic and post-embryonic phases.

## 1. Introduction

The requirement for protein sources has increased alongside the growth in the global population, which therefore necessitates an increase in the industrial breeding and production of animals, whose meat or eggs are consumed by humans to meet their protein needs. Achieving increased economic efficiency is one of the primary goals of poultry farmers, in addition to producing healthy and high-quality food. Research and studies on egg nutrient content are important to improve the composition of the human diet. Because of the importance of egg nutrients, including the roles of egg minerals on growth, health, and poultry production, recently, more research on this topic has been conducted [1,2,3]. Minerals are transferred and stored in eggs in two ways, including: (1) via the ovary to the egg yolk and (2) through the fallopian tube to the albumin, shell and shell membrane of egg [4]. Eggs contain approximately 60% albumin, 30% yolk and 10% shell. Egg yolk also contains 50% water, 33% fat and 17% protein [5], all of which can be used by the embryo [6]. Egg albumin, which provides the protein needed by the embryo, contains about 88% water and is the main source of sodium (Na) and potassium (K); however, it contains low levels of Fe, Cu, Mn and Zn [7,8]. Additionally, eggshell contains high amounts of minerals [9], including calcium (Ca), and lower amounts of iron (Fe), magnesium (Mg), manganese (Mn), phosphorus (P), zinc (Zn) and strontium (Sr). Of these minerals, a large amount of Ca and a lower content of Mg, and very small amounts of Fe, Mn and P, are released from the shell and are available for the embryo [10,11]. The contents of albumin, yolk sac and amniotic fluid are mixed during incubation; in fact, eggshell contains elements that are important minerals for the embryo, but due to its structural and protective role, most of the mineral content is not available to the embryo, and a small element of shell Ca and Mg are released to it [12]. Mineral deficiencies during the embryonic period can lead to skeletal and cardiovascular disease, as well as weakened immune systems. Minerals also contribute to the activity of enzymes related to the formation of skeletal system. Knowing the role of the shell in the mineral supply of the embryo makes it possible to better understand mineral metabolism in the embryo. As previously mentioned, the egg yolk is the main part of the embryo’s mineral storage, and information on mineral deposits and their usage by the embryo is important [13,14]. In comparison to the yolk, the eggshell releases a small amount of P, Fe and Mn, and studies performed on embryo yolk sacs suggest that the eggshell is a weak source of these minerals, which are used in different incubation periods. After hatching, chicks use the remaining yolk sac nutrients until they receive sufficient nutrients from their diet [14]. The quality and quantity of minerals at the time of ovulation, as well as identifying the period of the embryo’s use of these minerals, are vital factors in successful hatching, which is indicated by the increase or decrease in each of these elements during the incubation period. So, the purpose of the current study was to investigate the changes in some mineral values, including Ca, Cu, Fe, K, Mg, Mn, Na, P, Sr and Zn, in the yolks of fertile goose eggs during the incubation period.

## 2. Materials and Methods

### 2.1. Treatment

A total of 200 healthy and clean fertile goose eggs were selected and weighed separately. In this study, the incubation times were considered the experimental treatment. Sampling was performed on days 0 (day of setting), 9.5, 19.5, 25 and 30 (day of hatching) of incubation. The experimental design was completely randomized, with 5 treatments (incubation times) and 6 replicates for each treatment. During each time, 30 eggs were sampled and subdivided into 6 separate replicates of 5 eggs. Sampling was performed by creating a small hole at the larger end of the egg (where the air sac is located) and the yolk with the yolk sac membrane was separated after embryo removal. The yolks of 5 eggs in each replicate were mixed and considered one experimental replicate. The obtained samples were kept at −20 °C until laboratory analysis (mineral and fat analysis).

### 2.2. Incubation

Ten hours before incubation, the egg storage room temperature was set to 25 °C. A total of 200 fertile eggs were incubated in a standard incubator (37.2 to 37.5 °C and 65 to 70% RH) and a hatchery (37.0 to 37.2 °C and 95 to 100% RH). The eggs were rotated 360 degrees every 6 h.

### 2.3. Mineral Analysis

The concentrations of yolk minerals, including Ca, Fe, K, Mg, Mn, Na, P, Sr and Zn, were measured at each incubation time using an optical spectroscope. The samples were homogenized, weighed (as-is and dry weight) and freeze dried, and then, the concentrations of Ca, Cu, Fe, Mg, Mn, Na, K, Sr and Zn were measured. After powdering the dry samples, 0.5 g of each sample was placed in a 50 mL tube, and 5 mL of nitric acid was added. The samples were covered with a watch glass and heated in an oven at 50 °C for 2 h; then, the temperature was increased to 90 °C for 30 min. After cooling to room temperature, 2 mL of hydrogen peroxide was added. The samples were then heated again for 10 min at 90 °C; then, the temperature was raised to 120 °C for 30 min, and finally, the samples were diluted with 30 mL of distiller water. The prepared yolk samples were analyzed for mineral concentration using inductively coupled plasma optical emission spectroscopy (iCAP) according to manufacturer’s instructions (Agilent 700 Series ICP Optical Emission Spectrometers) [13]. From this, measured concentrations of these minerals were obtained. The yolk mineral concentrations were multiplied by the dried yolk weight to determine the absolute amount of mineral in each yolk. The dry yolk weight was subtracted from the fresh yolk weight to give the approximate water content.

### 2.4. Statistical Analysis

The whole data set was tested for normal distribution and variance homogeneity (Shapiro–Wilk). The data set was subjected to analysis of variance (ANOVA) using a General Linear Model (GLM) in SAS [15] software (version 13.2, SAS Institute Inc., Cary, NC, USA), according to the following model:

y_ij_ = μ + α_i_ + T_j_ + ε_ijk_,

where y_ijk_ represents all parameters as dependent variables; μ is the mean; α_i_ is the replicate random effect, T represents the effect of the jth incubating time (j = 1, … 5), and ε_ijk_ is the error. Subsequently, a Tukey test for repeated measures was carried out to evaluate the differences between the means during the incubation time. All means were expressed as square means and mean standard error. The significance level was set to *p* < 0.05.

## 3. Results

The concentration (mg/kg of egg) and absolute weight (mg or µg) of minerals in the goose egg yolks on days 0, 9.5, 19.5, 25 and 30 of incubation are presented in Table 1 and Table 2, respectively. Except for Mn, all the minerals measured in the goose egg yolks were affected by incubation time (*p* < 0.05).

The concentration of yolk Ca gradually increased with increasing incubation time (*p* < 0.0001). The increased Ca concentration in the first 9.5 days (0 to 9.5 d) was only 13.5% (2610.9 vs. 2964.1 mg/kg); however, the increase in the last 5 days (25 to 30 d) was 41.3% (6608.4 vs. 9339.5 mg/kg), indicating a supply of embryo Ca from other sources (Table 1). Incubation time’s effect on the absolute weight of Ca content in the yolks followed another trend. The Ca content of the yolks from days 0 to 9.5 of incubation significantly decreased (*p* < 0.0001), and then, gradually increased until day 30 (*p* < 0.0001, Table 2).

The concentration of yolk Cu on day 9.5 of incubation was statistically significantly higher than that of day 0 (*p* < 0.004), but on days 19.5, 25 and 30, it was not different from the first day of incubation (Table 1). Almost the same trend was followed regarding the absolute weight of yolk Cu, and the highest weight of yolk Cu occurred on day 9.5 of incubation (*p* < 0.0001); however, the weight of Cu on days 19.5, 25 and 30 was statistically significantly lower than on the first day of incubation (Table 2).

Unlike Ca, Fe concentration gradually decreased with increasing incubation time (291.48 mg/kg on day 0 to 88.13 mg/kg on day 30) (*p* < 0.0001, Table 1). A demonstrated trend was that the Fe content of the yolks gradually decreased with increasing incubation time, as well (8.33 mg/kg on day 0 to 1.19 mg/kg mg/kg on day 30) (*p* < 0.0001, Table 2).

The K concentration of the yolks increased until day 9.5 (*p* < 0.0001), and then, gradually decreased until day 30 of incubation; in this way, the K concentration on day 30 was statistically significantly lower than on the first day of incubation (*p* < 0.0001, Table 1). The absolute weight of K followed the same trend, as well, and the highest level of yolk K occurred on day 9.5 of incubation (107.4 mg) (*p* < 0.0001, Table 2).

As shown in Table 1, the concentration of yolk Mg gradually decreased until day 25 (360.65 to 145.99 mg/kg) of incubation (*p* < 0.0001), and then, remained unchanged until day 30 of incubation. Additionally, the absolute weight of Mg followed the same trend, decreasing from 10.30 mg on day 0 to 2.06 mg on day 25 of incubation, and remained unchanged until day 30 (Table 2).

The concentration of yolk Mn was not changed during the incubation period; however, on the initial days of incubation (0–9.5 d) it was numerically higher than the other times (Table 1). Incubation time’s effect on the absolute weight of Mn content in the yolks followed another trend (Table 2), in which the Mn content was unchanged until day 9.5 of incubation, and then, decreased until day 19.5 (57.56 to 27.74 µg) (P = 0.0028). Additionally, the Mn content remained unchanged from days 19.5 to 30 of incubation.

In the first 9.5 days of incubation, the Na concentration of the yolks from 1163.17 on day 0 reached 3129 mg/kg, and then, gradually decreased over time to 881 mg/kg on day 30 (*p* < 0.0001, Table 1). The absolute Na content followed the same trend. From days 0 to 9.5 of incubation, the absolute weight of yolk Na increased, and then, gradually decreased until day 30 of incubation (*p* < 0.0001, Table 2).

In contrast with Ca, the concentration of yolk P gradually decreased (7207.6 to 4784 mg/kg) with incubation time (*p* < 0.0001), indicating the increased P requirement of embryos with age (Table 1). This trend was also repeated in yolk P absolute weight, in which the weight of P gradually decreased until day 30 of incubation (*p* < 0.0001, Table 2).

The concentration of Sr gradually increased with incubation time; however, this increase was sharper between 9.5 and 19.5 days of incubation (11.52 to 14.93 µg/kg) (*p* < 0.0001, Table 1). The absolute weight of yolk Sr gradually decreased until day 19.5 of incubation (*p* < 0.05), and then, remained unchanged from days 19.5 to 30 of incubation (Table 2).

The concentration of yolk Zn decreased (about a two-fold decrease) from days 0 to 19.5 of incubation, and then, increased until day 25; then, it sharply decreased (about a three-fold decrease in the last 5 days) until hatching (day 30 of incubation) (*p* < 0.0001, Table 1). The absolute weight of Zn followed the same trend, as well (*p* < 0.0001, Table 2).

## 4. Discussion

As shown in the experiment, goose chicks hatch after 30 days of incubation, and the concentrations of nutrients (such as minerals) change during this time. It is important to understand some physiological changes that occur during chick incubation and during the chick’s sojourn within an egg. In this period, some minerals are transferred between the embryo and other parts of the egg (yolk, albumen and shell) [4]. The concentrations and absolute mineral content of yolk minerals during incubation reflect the embryo’s evolutionary process.

As mentioned above, the goal of the current study was to investigate the changes in the Ca, Cu, Fe, K, Mg, Mn, Na, P, Sr and Zn content of the yolks of fertile goose eggs during the incubation period; except for Ca, the yolk content of all the measured minerals on hatching day were statistically lower than on the first day of incubation. In the present study, P concentration was slightly lower, and Fe (about 150%), Mg (35%), and Na, Sr and Zn (17, 74 and 28%, respectively) were higher than the results reported by other authors in breeder hen egg [14], while the concentrations of Ca, Cu, Mn and K were in line with their findings [14]. The authors examined the mineral content of fertile egg yolk on days 0 (day of setting), 11, 13, 15, 17, 19, 20 and 21 (day of hatch), and found that the concentration of yolk P, Fe, Mn, Cu and Zn decreased by day 17 of setting, indicating the absorption of these minerals by the embryos. Our findings on the P concentration of goose egg yolk were in line with Yair and Uni [14]’s report on hen eggs, in which the concentration of yolk P decreased constantly. The results of their study [14] indicate that most of the consumption of yolk minerals by embryos occurs until day 17 of incubation. However, in contrast with our study, in Yair and Uni [14]’s work on hen eggs, Mn concentration was reduced during incubation time.

The results of the present study on day 25 of incubation show that the concentration of Ca was 6% lower, Cu was 78% higher, Fe was 70% higher, K was 93% percent lower, Mg was 135% lower, Mn was 18% lower, Na was 36% lower, P was 31% lower, Sr was 72% higher and Zn was 32% higher than the concentration measured on the same day of incubation in chickens (day 17.5) [13]. These differences may be due to the different species of birds involved in the studis. Previous reports indicated Cu, Fe, P and Zn as the elements that are found mainly in the yolk, and that both the concentration and absolute amounts of these elements decreased over time [16,17]. A change in yolk mineral absorbance was shown during different incubation times. For example, the absolute weight of yolk Zn decreased between days 19.5 and 25 days of incubation. This decrease may be due to a decrease in Zn transporters in the yolk membrane (ZnT-1) during this time [18]. The main source of egg Na and K is albumin. According to a previous report, both the concentration and absolute amounts of yolk Na and K are shown to increase in the first week of incubation [14]. Albumin, water, sodium and potassium are absorbed from the blastoderm through the surface of the ectoderm and transferred to the yolk through the surface of the endoderm [19,20]. Therefore, the first week of incubation can be advantageous for intra-embryonic injection in eggs that have been deficient in nutrients, especially minerals. This period of albumen influx ensures adequate yolk Na, K and water reserves for embryonic utilization during incubation and after hatching. This period of albumen influx could potentially provide an advantage in planning ovo injections on day 0 if eggs are known to be lacking in minerals or other nutrients.

As the results of the present study demonstrate, the absolute amount of Ca decreased from days 0 to 9.5 and increased until incubation. There are some reports indicating the transfer of Ca and Mg from the eggshell to the embryo through the chorioallantoic membrane during incubation [21,22,23,24]. Additionally, the increased yolk Sr may be due to the transfer of the element from the eggshell to the embryo through the chorioallantoic membrane. The Sr content of eggshell can be increased using dietary supplementation [25]. The dietary supplementation of 1000 mg/kg of Sr in the poultry diet results in a decrease in the Mg content of the eggshell. However, the supplementation of 500 mg/kg of Sr does not have a significant effect on eggshell Mg content [25]. The dietary supplementation of Sr improves growth performance and bone mineralization in broiler chickens [26]. The Mg content of eggshells can be increased by dietary Mg supplementation [27]. The Sr content of egg yolk can be increased by dietary supplementation by improving the availability of this element in the eggshell. Supplying a balanced amount of Mg and Se has beneficial effects on growth performance and bone mineralization in broiler chickens; however, excess of these minerals has a negative effect on the absorption of Ca and P [28]. Mg is mainly found in egg yolks, and the amount of this element in eggshells is poor [14]. Mn was the only element that did not show significant changes during incubation, but its absolute amount decreased over time. By examining the number of changes in the minerals mentioned, we obtain their place on the periodic table. Na and K, which had a similar trend during incubation, and were transferred from the albumin to the the yolk, are members of the alkaline group. Mg, Ca and Sr, which are secreted from the shell to the yolk during incubation, are also alkali metals. Cu, Fe, Mn and Zn are the transition metals of group 4 of the periodic table, and P is a non-metallic element that iwass reduced from days 0 to 18 of incubation; this is due to the increased uptake of this element by the embryo, because P is an important component of phospholipids, proteins and nucleic acids [29], which are utilized by embryos to develop essential organs in the body, including the brain. Our results on egg yolk P changes agree with Hopcroft et al. [13].

Richards [30] examined the mineral concentration of turkey egg yolk in ex ovo (shell-less culture) and in ovo incubated embryos [30], and showed that the concentrations of Zn, Cu and Fe in in ovo-incubated embryos decreased, while the concentrations of Ca and Mn were enhanced. However, in ex ovo embryo yolks, the aforementioned minerals followed another trend. In ex ovo embryo yolks, the increase in Mn and Ca was lower than in ovo embryo yolks, and the concentrations of Zn, Cu and Fe were not affected by the incubation period.

Yolk Ca content is regulated through two processes: (1) the absorption of Ca to the embryonic circulation from the yolk (which decreases the yolk Ca concentration), and (2) the mobilization of Ca from the eggshell into the yolk via the chorioallantoic membrane (which enhances yolk Ca concentration) [30,31]. As a result of the enhanced Ca concentration in the second half of incubation, it can be concluded that Ca deposits from the eggshell into the yolk are higher than Ca uptake by the embryonic circulatory system during this period.

Some authors evaluated the effect of breeder birds’ age on the mineral content of egg yolk (32). It was shown that transfer of P, K, Ca, Mg, Cu, Fe, Mn and Zn from breeder hens to egg yolks was affected by breeder age, and the content of the previously stated minerals in egg yolks from 32-week-old birds was higher than that of older birds (42 and 52 weeks old) [32]. The egg yolks of young breeders (28 weeks old) contained more minerals than those of older breeders (34 and 40 weeks old) in ducks [33]. As probably the first study looking at the yolk mineral content of goose eggs, we could not find any other reports with which to compare our results. There seems to be a large difference in the decrease and increase in minerals mentioned in poultry species during incubation; however, the trend of decreasing or increasing in some minerals is consistent across each species. Additionally, some minerals differ between species.

On day 25 of incubation, Sr numerically increased compared to day 19.5. As the shell contains Sr [34], the increase in yolk Sr during this same period may be due to mobilization of shell Sr by the chorioallantoic membrane, and its subsequent deposition in the yolk. Manganese is predominately found in the yolk; however, it is also present in the shell [14]. Manganese yolk concentration did not significantly change throughout day 19.5 of incubation; however, absolute yolk Mn decreased over time. Relatively little is known about Mn storage in the yolk or the transport of Mn through the yolk sac membrane [30], and research designed to understand the usage and storage of embryonic yolk Mn may reveal unexpected mechanisms at work [13].

Interestingly, the grouping of the minerals outlined above corresponds to the locations of the elements on the periodic table. Potassium and Na, undertaking albumen efflux, are both in the alkali group; Mg, Ca and Sr, which are mobilized from the shell, are in the alkaline earth metals group; Cu, Fe, Mn and Zn are group 4 transitional metals, and P is a non-metal. This observation may be relevant to ion transportation across the yolk sac membrane [13].

## 5. Conclusions

As per the obtained results of the current study, due to the ability to alter mineral levels during incubation, there is an opportunity to supply sufficient levels of these minerals to meet the needs of embryos. For these reasons, further studies must be conducted in order to understand how to supply the necessary minerals during the incubation period of eggs.

## Figures and Tables

**Table 1:** Concentrations of goose egg yolk minerals (±SD) at different incubation times.

Mineral (mg/kg)	Embryonic Days					
	0	9.5	19.5	25	30	*p*-Value
Ca	2610.9 ± 159 ^e^	2964.1 ± 140 ^d^	5149.3 ± 139 ^c^	6608.4 ± 118 ^b^	9339.5 ± 270 ^a^	<0.0001
Cu	4.91 ± 0.22 ^b^	9.06 ± 2 ^a^	5.48 ± 0.66 ^b^	6.32 ± 1.48 ^b^	5.80 ± 1.36 ^b^	<0.0040
Fe	291.48 ± 9.75 ^a^	262.72 ± 1.94 ^b^	191.72 ± 3.79 ^c^	129.72 ± 28 ^d^	88.13 ± 23 ^e^	<0.0001
K	1639.9 ± 18.16 ^c^	2463.1 ± 199 ^a^	2156.3 ± 76 ^b^	1211.2 ± 256 ^d^	1010.7 ± 93 ^d^	<0.0001
Mg	360.65 ± 40.90 ^a^	290.22 ± 7.12 ^b^	244.20 ± 39 ^c^	145.99 ± 24 ^d^	162 ± 10.7 ^d^	<0.0001
Mn	2.22 ± 0.95 ^a^	2.68 ± 0.81 ^a^	1.80 ± 0.09 ^a^	1.76 ± 0.34 ^a^	1.86 ± 0.36 ^a^	<0.2143
Na	1163.17 ± 67.77 ^bc^	3129 ± 59 ^a^	1243.52 ± 153 ^b^	1039.98 ± 104 ^c^	881 ± 65.26 ^d^	<0.0001
P	7207.6 ± 200 ^a^	6148.5 ± 165 ^b^	5700.4 ± 96 ^c^	5803.9 ± 243 ^c^	4784 ± 69 ^d^	<0.0001
Sr	10.18 ± 2 ^b^	11.52 ± 1.33 ^b^	14.93 ± 0.54 ^a^	16.47 ± 1.23 ^a^	16.42 ± 0.94 ^a^	<0.0001
Zn	94.04 ± 1.14 ^a^	88.36 ± 1.02 ^b^	45.56 ± 3.37 ^d^	70.36 ± 2.55 ^c^	25.97 ± 5.16 ^e^	<0.0001

Means within a row with different superscripts significantly differ (*p* < 0.05).

**Table 2:** Absolute weights of goose egg yolk minerals (±SD) at different incubation times.

Mineral	Embryonic Days					
	0	9.5	19.5	25	30	*p*-Value
Ca (mg)	74.60 ± 4.54 ^c^	63.62 ± 3.02 ^d^	79.26 ± 2.15 ^c^	93.48 ± 1.68 ^b^	126.14 ± 3.64 ^a^	<0.0001
Cu (µg)	143.35 ± 6.36 ^b^	194.5 ± 43 ^a^	84.31 ± 10 ^c^	89.43 ± 21 ^c^	78.39 ± 18 ^c^	<0.0001
Fe (mg)	8.33 ± 0.27 ^a^	5.64 ± 0.04 ^b^	2.95 ± 0.06 ^c^	1.83 ± 0.40 ^d^	1.19 ± 0.31 ^e^	<0.0001
K (mg)	50.73 ± 0.56 ^c^	107.4 ± 8.7 ^a^	71.75 ± 2.55 ^b^	33.46 ± 7.09 ^d^	17.41 ± 1.61 ^e^	<0.0001
Mg (mg)	10.30 ± 1.16 ^a^	6.23 ± 0.15 ^b^	3.76 ± 0.61 ^c^	2.06 ± 0.34 ^d^	2.19 ± 0.14 ^d^	<0.0001
Mn (µg)	63.57 ± 27 ^a^	57.56 ± 17 ^a^	27.74 ± 1.42 ^b^	24.94 ± 4.87 ^b^	25.18 ± 4.94 ^b^	<0.0028
Na (mg)	33.251.93 ^b^	67.16 ± 1.27 ^a^	19.14 ± 2.36 ^c^	14.71 ± 1.48 ^d^	11.90 ± 0.36 ^e^	<0.0001
P (mg)	205.93 ± 5.73 ^a^	131.97 ± 3.54 ^a^	87.74 ± 1.49 ^c^	82.1 ± 3.44 ^d^	64.61 ± 0.93 ^e^	<0.0001
Sr (µg)	290.95 ± 57 ^a^	247.28 ± 28 ^ab^	229.83 ± 8.37 ^b^	232.98 ± 17 ^b^	221.78 ± 12 ^b^	<0.0408
Zn (mg)	2.68 ± 0.03 ^a^	1.9 ± 0.02 ^b^	0.70 ± 0.05 ^d^	0.99 ± 0.03 ^c^	0.35 ± 0.06 ^e^	<0.0001

Means within a row with different superscripts significantly differ (*p* < 0.05).

## Data Availability

Not applicable.
